# Antibacterial resistance and their genetic location in MRSA isolated in Kuwait hospitals, 1994-2004

**DOI:** 10.1186/1471-2334-6-168

**Published:** 2006-11-25

**Authors:** Edet E Udo, Noura Al-Sweih, Eiman Mokaddas, Molly Johny, Rita Dhar, Huda H Gomaa, Inaam Al-Obaid, Vincent O Rotimi

**Affiliations:** 1 Department of Microbiology, Faculty of Medicine, Kuwait University, Kuwait; 2 Microbiology Laboratories, Department of Laboratory Medicine, Ministry of Health, Kuwait

## Abstract

**Background:**

Methicillin-resistant *Staphylococcus aureus *(MRSA) continues to be a major cause of serious infections in hospitals and in the community worldwide. In this study, MRSA isolated from patients in Kuwait hospitals were analyzed for resistance trends and the genetic location of their resistance determinants.

**Methods:**

Between April 1994 and December 2004, 5644 MRSA isolates obtained from different clinical samples were studied for resistance to antibacterial agents according to guidelines from the National Committee for Clinical Laboratory Standards and the British Society for Antimicrobial Chemotherapy. The genetic location of their resistance determinants was determined by curing and transfer experiments.

**Results:**

They were resistant to aminoglycosides, erythromycin, tetracycline, trimethoprim, fusidic acid, ciprofloxacin, chloramphenicol, rifampicin, mupirocin, cadmium acetate, mercuric chloride, propamidine isethionate and ethidium bromide but susceptible to vancomycin, teicoplanin and linezolid. The proportion of the isolates resistant to erythromycin, ciprofloxacin and fusidic acid increased during the study period. In contrast, the proportion of isolates resistant to gentamicin, tetracycline, chloramphenicol and trimethoprim declined. High-level mupirocin resistance increased rapidly from 1996 to 1999 and then declined. They contained plasmids of 1.9, 2.8, 3.0, 4.4, 27 and 38 kilobases. Genetic studies revealed that they carried plasmid-borne resistance to high-level mupirocin resistance (38 kb), chloramphenicol (2.8 – 4.4 kb), erythromycin (2.8–3.0 kb) and cadmium acetate, mercuric chloride, propamidine isethionate and ethidium bromide (27 kb) and chromosomal location for methicillin, the aminoglycosides, tetracycline, fusidic acid, ciprofloxacin and trimethoprim resistance. Thus, the 27 kb plasmids had resistance phenotypes similar to plasmids reported in MRSA isolates in South East Asia.

**Conclusion:**

The prevalence of resistance to erythromycin, ciprofloxacin, high-level mupirocin and fusidic acid increased whereas the proportion of isolates resistant to gentamicin, tetracycline, chloramphenicol and trimethoprim declined during the study period. They contained 27-kb plasmids encoding resistance to cadmium acetate, mercuric chloride, propamidine isethionate and ethidium bromide similar to plasmids isolated in MRSA from South East Asia. Molecular typing of these isolates will clarify their relationship to MRSA from South East Asia.

## Background

Methicillin-resistant *Staphylococcus aureus *(MRSA) has continued to be a major pathogen causing infections in hospitals and in the community and are increasingly isolated in hospitals worldwide. Following its initial isolation in the UK in 1961, many outbreaks of infections due to MRSA have occurred and it has now become endemic in several centers in the world [1-3]. MRSA were initially associated with large teaching hospitals, but they now also colonize or cause infections in patients in smaller health care facilities, nursing homes, general hospitals [2,3] and in the community [3-6]. MRSA are important because, in addition to being methicillin or oxacillin- resistant, most of them are also resistant to commonly used antibiotics. There is evidence that antibacterial resistance is associated with an increased length of hospital stay, increased financial burden [7] and increased mortality [8] associated with infection in the hospitalized patient. Therefore, knowledge of the susceptibility patterns of local strains is essential for the judicial use of antibacterial agents for empiric therapy

Some MRSA strains, known as epidemic MRSA (EMRSA), can spread rapidly between patients within and between hospitals thereby causing major problems for infection control [1-3,9,10]. The incidence of MRSA differs in different hospitals, states or countries. Even within the same hospital, their incidence may differ between wards. MRSA also differ in their resistance to antibacterial agents and in the genetic location of these resistance determinants. Studies have shown that the genetic determinants for antibiotic resistance reside on plasmids, chromosomal DNA or on transposable elements [11,12]. The acquisition or loss of these genetic determinants may contribute to changes in the resistance patterns in a particular environment. Changes in resistance patterns can also be due to the introduction of different resistant clones to a healthcare facility by healthcare workers or patients [13]. In this report, we present the results of studies conducted on MRSA isolated from Kuwait hospitals from 1994 to 2004 for their resistance patterns and the genetic location of their resistance determinants.

## Methods

### MRSA isolates

The MRSA isolates for this study were submitted for typing to the MRSA Reference Laboratory situated at the Department of Microbiology, Faculty of Medicine, Kuwait University, Kuwait. A total of 5,644 consecutive MRSA isolates were received for typing and antibiotic susceptibility testing between 1994 and 2004. They were identified by cultural characteristics, Gram's stain, and positive tube coagulase and DNase tests in the individual laboratories and pure cultures were sent to the reference laboratory for *mecA *PCR and typing. The isolates were preserved in glycerol 15% (v/v) in brain heart infusion broth (BHIB, Oxoid, Basingstoke, UK) at -80°C. They were recovered by subculturing in BHIB at 35°C for 24 hr followed by two further subcultures on brain heart infusion agar. This study was performed with funds from projects MI 088, MI 091 and MI 01/03 from Kuwait University Research Administration. This study did not require the approval of the university ethics committee.

### Susceptibility to antimicrobial agents

Susceptibility to antimicrobial agents was determined by the disk diffusion method [14] on Mueller-Hinton Agar (Oxoid, UK) incubated for 24 hr at 35°C. The following antibiotic disks (Oxoid) were used: oxacillin (1 μg), benzyl penicillin (2U), kanamycin (30 μg), neomycin (30 μg), streptomycin (30 μg), tobramycin (10 μg), mupirocin (200 μg), gentamicin (10 μg), erythromycin (15 μg), chloramphenicol (30 μg), tetracycline (10 μg), trimethoprim (2.5 μg), fusidic acid (10 μg), rifampicin (5 μg), ciprofloxacin (5 μg), teicoplanin (30 μg), vancomycin (30 μg), linezolid (30 μg). Susceptibility to fusidic acid was performed according guidelines by the British Society for Antimicrobial Chemotherapy [15]. For susceptibility to non antibiotics, disks containing the agents were prepared in the laboratory with the following concentrations: cadmium acetate (50 μg), mercuric chloride (109 μg), propamidine isethionate (100 μg) and ethidium bromide (120 μg) [16].

Minimum inhibitory concentration (MIC) of oxacillin, mupirocin, vancomycin and teicoplanin were determined using E-test strips (AB Biodisk, Solna, Sweden) according to the manufacture's instructions. Methicillin resistance was confirmed by detecting the amplification of the *mecA *gene from 1994–2000 and by the detection of PBP 2a gene product using a rapid latex agglutination kit (Denka-Seiken, Japan) according to the manufacturer's instruction from 2001 to 2004.

### Amplification of the *mecA *gene

The *mecA *gene for methicillin resistance was amplified using primers purchased from Gibco BRL (Life Science Technologies, UK). The description of the primers and methods have been published previously [17].

### Genetic location of resistance determinants

Plasmids were isolated by the cetyltrimethylammonium bromide method as described previously [18] and separated by agarose gel electrophoresis. The genetic location of the resistance determinants was studied by curing and transfer experiments as described previously [18].

For curing of resistance and plasmids, the isolates were incubated on BHIA at 45°C for 48 hr and single colonies of each isolate were screened by replica plating for the loss of resistance. At least 300 colonies were screened for each isolate. Loss of resistance was confirmed by disk diffusion tests. Colonies that lost antimicrobial resistance were screened for plasmid loss by agarose gel electrophoresis. For the transfer of resistance and plasmids, representative isolates were selected based on their resistance and plasmid profiles and used as donors in conjugation and mixed-culture transfer (MCT) experiments using strains WBG541 and WBG1876 as recipients respectively as described previously [18]. Selections for transconjugants and transcipients were made on brain heart infusion agar containing (mg/L) fusidic acid (5), rifampicin (2.5) and one of the following agents: tetracycline (10), erythromycin (5), Chloramphenicol (10), mupirocin (10), trimethoprim (5), cadmium acetate (60), propamidine isethionate (100). Transconjugants or transcipients were screened for plasmid carriage by agarose gel electrophoresis.

## Results

A total of 5,644 MRSA isolates were tested from 14 hospitals in Kuwait between 1994 and 2004. The number of isolates received yearly varied from 342 in 1994 to 930 in 2004. This was due to the increase in the number of laboratories submitting MRSA for typing from four in 1994 to 14 in 2004. All of them contained the *mecA *DNA and were resistant to the agents summarized on Table 1 but were all susceptible to vancomycin, teicoplanin and linezolid.

**Table 1:** Antibacterial resistance of MRSA isolates, 1994–2004.

Year	Antibacterial resistance in MRSA (%)												
	
	#	**Gm**	**Km**	**Sm**	**Em**	**Cm**	**Tet**	**Tp**	**Cip**	**Fa**	**Rf**	**MupH**	Cd*
1994	342	98	100	100	66	25	94	86	53	22	1.0	ND	100
1995	298	95	98	100	89	25	92	87	23	45	1.4	ND	98
1996	421	100	100	100	68	44	94	93	41	32	3.0	6.0	100
1997	245	94	99	99	81	23	95	75	72	57	5.0	17	97
1998	329	88	95	77	74	21	92	61	63	75	3.0	24	94
1999	336	84	92	92	69	4.0	93	38	79	89	2.0	28	94
2000	542	86	95	94	76	3.0	89	34	72	81	8.0	22	98
2001	533	77	88	89	86	2.0	79	37	81	88	1.0	2.7	94
2002	883	85	95	89	88	4.0	90	16	96	90	5.0	3.0	93
2003	785	77	88	89	86	4.0	88	17	88	85	1.0	1.0	91
2004	930	81	89	86	88	2.0	88	27	92	92	4.7	4.7	89

**Abbreviations**: #, number of isolates Gm, gentamicin, Km, kanamycin, Sm, streptomycin, Em, erythromycin, Cm, chloramphenicol, Tet, tetracycline, Tp, trimethoprim, Cip, ciprofloxacin, Fa, fusidic acid, Rf, rifampicin, MupH, high-level mupirocin resistance, Cd, cadmium acetate, also resistant to mercuric chloride, propamidine isethionate and ethidium bromide, ND, not detected

Rifampicin resistance remained low throughout the study period with a small increase observed in 2000. In contrast, resistance to fusidic acid, ciprofloxacin and erythromycin showed increasing trends. The prevalence of fusidic acid resistance increased from 22% in 1994 to 92% in 2004. The prevalence of erythromycin resistance increased from 66% in 1994 to 88% in 2004. The proportion of MRSA isolates resistant to ciprofloxacin increased from 53% in 1994 to 92% in 2004 after a peak of 96% in 2002. On the contrary, resistance to chloramphenicol, trimethoprim, gentamicin, kanamycin, streptomycin, tetracycline showed decreasing trends. The prevalence of trimethoprim resistance declined from 86% in 1994 to 27% in 2004. Chloramphenicol resistance decreased from 25% in 1994 to 2% in 2004. The proportion of isolates resistant to gentamicin decreased from 98% in 1994 to 77% in 2001 before increasing to 81% in 2001. Similarly, the proportion of isolates resistant to kanamycin and streptomycin decreased although they were still above 80 percent. High level mupirocin resistance increased from 6% in 1996 and peaked at 28% nationally in 1999 before it declined to 1.0 % in 2003 and increased again to 4.7% in 2004. The prevalence of high -level mupirocin resistance was higher in some hospitals, especially at the Burn unit where it had reached 56% in 1997 [16]. Resistance to cadmium acetate, mercuric chloride, propamidine isethionate and ethidium bromide remained high with slight annual fluctuations.

### Susceptibility to vancomycin and teicoplanin

Following the reports of vancomycin intermediate resistance in MRSA in 1997 [19], vancomycin and teicoplanin MIC were determined for all MRSA isolates submitted to the typing laboratory from 1997. All of the isolates examined from 1997 to 1998 had vancomycin and teicoplanin MIC values of 0-5-2 mg/L. However, from 1999, reduced susceptibility to both vancomycin and teicoplanin (MIC: 3–4 mg/L) started to appear. The results of the isolates tested between 1999 and 2004 are summarized in Table 2.

**Table 2:** Susceptibility of MRSA isolates to vancomycin and teicoplanin

			Vancomycin MIC (%)			Teicoplanin MIC (%)		
S/N	Year	#	≤ 2 mg/L	3 mg/L	4 mg/L	≤ 2 mg/L	3 mg/L	4 mg/L

1	1999	336	199(59.2)	120(35.7)	17(5.1)	225(70.0)	99(29.5)	12(3.5)
2	2000	542	494(91.1)	47(8.7)	1(0.2)	479(88.4)	58(10.7)	5(0.9)
3	2001	533	385(72.2)	133(25.0)	15(2.8)	293(55.0)	160(30.0)	80(15.0)
4	2002	884	773(87.4)	107(12.1)	4(0.5)	740(83.7)	112(12.7)	32(3.6)
5	2003	785	653(83.2)	132(16.8)	0.00	623(79.4)	162(20.6)	0.00
6	2004	930	807(86.8)	121(13.0)	2(0.2)	740(79.6)	176(18.9)	14(1.5)

Total		4010	3311(82.5)	660(16.5)	39 (1.0)	3100 (77.3)	767(19.1)	143(3.6)

### Plasmid analysis of MRSA isolates

**Figure 1:**
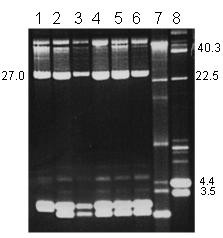
Plasmid content of mupirocin-susceptible MRSA, Lanes 1–6, representatives of multiresistant MRSA carrying the 27 kb plasmid encoding resistance to cadmium acetate, mercuric chloride, propamidine isethionate and ethidium bromide and the 2.8–3.0 kb plasmids. Lane 7, a representative of non multiresistant MRSA. Lane 8, *S. aureus *strain WBG4483 containing plasmid molecular weight standards. Sizes are in kb.

**Figure 2:**
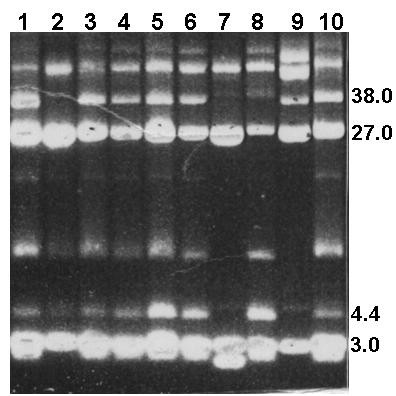
Plasmid contents of high-level mupirocin-resistantisolates Lanes 1, 3, 4, 5, 6, 9, 10 contained the 38-kb mupirocin resistance plasmid and 4.4 kb chloramphenicol resistance plasmid in addition to the 27 kb plasmids. Sizes are in kb.

Plasmid DNA was detected in all of the MRSA isolates. The plasmids ranged in size from *c*2.0 kb to 38.0 kb and the number of plasmids in each cell varied between two and four. The plasmid contents of representative MRSA isolates are presented in Figures 1 and 2. All carried a 27 kb plasmid. The isolates differed in the carriage of the 38.0, 3.0, 4.4 and 2.8 kb plasmids. Approximately 60 % of them contained three plasmids with sizes of 27, 3–4 and 2.8 kilobases (Figure 1), 30% of them contained two plasmids of 27 and 3–4 kilobases and approximately 10% of them carried four plasmids. The 38.0 kb plasmid was present only in isolates that expressed high-level mupirocin-resistance (Figure 2).

### Genetic location of antibacterial resistance determinants

Representative isolates having different resistance and plasmid patterns were selected and used in curing and transfer experiments to ascertain the genetic location of the different resistance determinants. In curing experiments, resistance to cadmium, mercuric chloride, propamidine isethionate and ethidium bromide were always lost together with the loss of the 27 kb plasmids. The loss of chloramphenicol resistance in chloramphenicol resistant isolates corresponded to the loss of 4.4 kb plasmids. The isolates were studied further by using them as donors in mixed-culture transfer and conjugation experiments to isolate and characterize their plasmids. Representatives of the plasmids and resistance transferred are presented in Table 3. In mixed-culture transfer experiments, resistance to chloramphenicol, erythromycin, cadmium acetate, mercuric chloride, propamidine isethionate and ethidium bromide were transferred. The transfer of resistance to cadmium acetate, mercuric chloride, propamidine isethionate and ethidium bromide was accompanied by the transfer of a 27 kb plasmid. Three different plasmid sizes were associated with chloramphenicol resistance. It was co – transferred with a 2.8 kb and a 3.5 kb plasmids in mupirocin-susceptible isolates and co transferred wit a 4.4 kb plasmid in the high-level mupirocin resistant isolates. In these isolates, the 4.4 kb plasmid could be transferred in both mixed-culture and conjugation experiments. The transfer of erythromycin resistance was accompanied by the transfer of 2.8 kb plasmid except in one isolate obtained in 1995 where erythromycin resistance was transferred together with a 3.0-kb plasmid. In some instances, erythromycin resistance was transferred but no plasmids were detected in the transcipients.

**Table 3:** Properties of plasmids isolated from MRSA isolates

**S/N**	**Plasmids**	**Size, kb**	**Resistance phenotype**	Transfer Mode*	***Eco*RI Restriction pattern**
1	pXU01	27.1	Cd,Hg,Pi,Eb	M	10.4, 6.4, 4.1, 2.6, 2.3, 1.3
2	pXU02	27.1	Cd,Hg,Pi,Eb	M	10.4, 6.4, 4.1, 2.6, 2.3, 1.3
3	pXU04	27.1	Cd,Hg,Pi,Eb	M	10.4, 6.4, 4.1, 2.6, 2.3, 1.3
4	pXU08	27.6	Cd,Hg,Pi,Eb	M	11.5, 6.4, 3.5, 2.6, 2.3, 1.3
5	pXU09	27.6	Cd, Hg,Pi,Eb	M	11.5, 6.4, 3.5, 2.6, 2.3, 1.3
6	pXU12	31.5	mupH	C	7.8, 6.6, 6.1,4.9, 2.7, 1.8, 1.6
7	pXU16	38.0	mupH	C, M	ND
8	pXU17	38.0	mupH	C,M	ND
9	pXU03	2.8	Cm	M	ND
10	pXU11	3.5	Cm	M	ND
11	pXU14	4.4	Cm	M, C	ND
12	pXU07	2.8	Em	M	ND
13	pXU13	3.0	Em	M	ND

Abbreviations: Cd, cadmium acetate, Cm, Chloramphenicol, Eb, ethidium bromide, Em, erythromycin, Hg, mercuric chloride, Pi, propamidine isethionate, mupH, high-level mupirocin, ND, not done.

*, M, mixed culture transfer; C, conjugation.

High-level mupirocin resistance was transferred in conjugation as well as in mixed-culture transfer experiments and its transfer was accompanied by the transfer of a 38 kb plasmid. Chloramphenicol resistance was co transferred with high-level mupirocin resistance in conjugation experiments whether the transconjugants were selected on plates containing mupirocin or chloramphenicol. Resistance to gentamicin, kanamycin, streptomycin, tetracycline, fusidic acid, trimethoprim and ciprofloxacin could not be transferred in either mixed-culture transfer or conjugation experiments.

### Restriction endonuclease digests of plasmids encoding resistance cadmium acetate, propamidine isethionate and ethidium bromide

The 27 kb plasmids were digested with *Eco*RI restriction endonuclease and their restriction patterns compared. The results presented in Table 3 showed that they were of two closely related types. The first type consisted of plasmids, pXU01, pXU02 and pXU04 with *Eco*RI restriction fragments sizes of 10.4, 6.4, 4.1, 2.6, 2.3 and 1.3 kilobases. The second type consisted of plasmids pXU08 and pXU09 that were isolated from isolates expressing high-level mupirocin resistance. They had *Eco*RI restriction fragment sizes of 11.5, 6.4, 3.5, 2.6, 2.3 and 1.3 kilobases. The two types differed only in the sizes of two fragments.

## Discussion

Methicillin – resistant *Staphylococcus aureus *continues to be a major cause of problems in hospitals and in the community [1-3,6]. Although several studies have documented the increasing prevalence of MRSA worldwide [20-26], reports documenting resistance trends in them are scanty [23,27]. This report details antibacterial resistance trends and their genetic determinants in MRSA isolated in Kuwait hospitals from 1994 to 2004.

Most of them were resistant to the aminoglycosides, macrolides, tetracyclines, trimethoprim, ciprofloxacin, fusidic acid, heavy metal ions and nucleic acid-binding compounds [16] with resistance profiles similar to the epidemic MRSA isolates from the UK (EMRSA1), Australia (EA MRSA), Singapore and Malaysia obtained in the late 1980s and early 1990s [12,28,29]. Although no intermediate vancomycin resistant isolate (MIC: 8–16 mg/L) was found during the study period, 17.5% and 22.7.1% of the isolates demonstrated reduced susceptibility to vancomycin and teicoplanin respectively (MIC: 3–4 mg/L) (Table 2). This is of concern and warrants closer observation to detect any further increases in glycopeptides MIC levels.

Analysis of the resistance patterns revealed interesting trends. While resistance to erythromycin, ciprofloxacin and fusidic acid increased, others such as chloramphenicol, trimethoprim, and gentamicin declined over time while the proportion of rifampicin-resistant isolates remained low. The proportion of MRSA isolates resistant to ciprofloxacin increased from 53% in 1994 to 92% in 2004. An increase in the proportion of MRSA isolates resistant to ciprofloxacin (from 4.9% in 1998 to 75.9% in 1999) was also observed in MRSA isolated in Australian teaching hospitals from1998 to1999 [27]. Similarly, a study of antibiotic resistance in UK hospitals also reported an increase in ciprofloxacin resistance from 14.9% to 23.8% in a 12 month period [30]. Furthermore, changes in the prevalence of trimethoprim resistance in this study were similar to that observed in the Australian study where trimethoprim resistance also declined from 98.4% in 1989 to 82.4% in 1999 [27].

Fusidic acid resistance showed an increasing trend with the proportion resistant isolates increasing from 22% in 1994 to 92% in 2004. These levels of fusidic acid-resistant MRSA in Kuwait hospitals are higher than what is reported in many centers worldwide [31-33]. Although fusidic acid resistance can arise readily by mutation in the laboratory, its incidence in clinical MRSA isolates is still relatively low despite increasing reports worldwide [31-33]. High levels of fusidic acid have been previously associated with areas of hospitals where cross infection is common suggesting the spread of a resistant clone [32]. A review of fusidic acid consumption in two Kuwait hospitals revealed that an average of 650 g of oral fusidic acid was consumed annually in these hospitals [34]. This may have contributed to the emergence of fusidic acid resistance in the first place. It was also demonstrated by pulsed-field gel electrophoresis that the fusidic acid -resistant MRSA in Kuwait hospitals belonged to few genetic backgrounds [34]. The transmission of these clones and their maintenance in the different hospitals may explain their high prevalence in Kuwait hospitals. Furthermore, the presence of fusidic acid resistance in both multiresistant and non multiresistant MRSA isolates this study supports the heterogeneous mode of acquisition of resistance.

Although the overall prevalence of gentamicin resistance was high throughout the study period, it declined over time from 98% in 1994 to 80% in 2004 (Table 1). This decline, which started in about 1998 appeared to have coincided with the emergence of non multiple resistant MRSA isolates including community -associated MRSA in Kuwait hospitals which was not fully appreciated till 2000 [35]. It is now well established that community-acquired MRSA is an increasing problem in the community as well as in hospitals [6,33,35].

High-level mupirocin resistance was first reported in MRSA isolated from a patient in a Burns unit in Kuwait five years after mupirocin was introduced for clinical use in Kuwait, in 1992 [36] due to the acquisition of a 38-kb conjugative plasmid encoding high level mupirocin resistance by a previously mupirocin-susceptible MRSA in the Burns unit. The high-level mupirocin-resistant MRSA clone then spread to other hospitals [37,38] and the proportion of high-level mupirocin resistant MRSA increased sharply from 6% in 1996 to 28% in 1999 and then declined to 4% in 2004 following the restriction of mupirocin use and the disappearance of the resistant clone. The prevalence of MRSA resistant to mupirocin has also been reported to decline following mupirocin withdrawal in a USA hospital [39].

Results of studies to determine the genetic location of the resistance determinants revealed that the multiresistant MRSA isolates carried plasmid-mediated resistance to chloramphenicol, high-level mupirocin, erythromycin, cadmium acetate, mercuric chloride, propamidine isethionate and ethidium bromide. Resistance to cadmium acetate, mercuric chloride, propamidine isethionate and ethidium bromide were linked and carried on closely related 27 kb plasmids (Table 3). These plasmids have resistance phenotypes similar to plasmids present in MRSA isolated in Singapore, Malaysia and other South East Asian countries [12,28,29] but differed from plasmids found in the classic MRSA isolated in the 1960 and1970s which carried plasmid-borne resistance to tetracycline, low level streptomycin resistance and penicillinase production [12]. These results indicated that resistance to methicillin, gentamicin, kanamycin, streptomycin; trimethoprim and ciprofloxacin were located on the bacterial chromosome similar to the Eastern Australian MRSA [27,29]. These results suggest that the most common MRSA types in Kuwait hospitals appear to be related to MRSA isolated in South East Asia [12,28]. Molecular typing with multi locus sequence analysis will help clarify this relationship.

## Conclusion

This study has demonstrated the changing prevalence of antibiotic resistance in MRSA in Kuwait and determined the genetic location of their resistance determinants. In addition, it has highlighted the different factors contributing to the high prevalence of MRSA in Kuwait. These factors include (i) the persistence of multiply resistant MRSA clones demonstrated by the high prevalence of multiply resistant isolates and their spread among the hospitals [38], (ii) the acquisition, transmission and subsequent loss of the 38-kb plasmid encoding high-level mupirocin resistance and (iii) the introduction and apparent transmission of non multiresistant MRSA including EMRSA-15 and community acquired MRSA in Kuwait hospitals [35]. Therefore efforts must continue to be exerted to minimize further selections and spread of new MRSA clones.

## Competing interests

The author(s) declare that they have no competing interests.

## Authors' contributions

EEU planned the study, analyzed results, compiled the results and wrote the manuscript. The coauthors, NA, EM, MJ, IA, RD, HG and VOR helped in planning the study, analyzed the susceptibility results from their hospitals and revised the manuscript.

## Pre-publication history

The pre-publication history for this paper can be accessed here:



## References

[B1] Brumfit W, Hamilton-Miller J (1989). Methicillin-resistant *Staphylococcus aureus*. N Eng J Med.

[B2] Boyce JM (1994). Methicillin-resistant *Staphylococcus aureus *: a continuing infection control challenge. Eur J Clin Microbiol Infect Dis.

[B3] Chambers HF (2001). The changing epidemiology of *Staphylococcus aureus*. Emerg Infect Dis.

[B4] Udo EE, Pearman JW, Grubb WB (1993). Genetic analysis of community isolates of methicillin-resistant *Staphylococcus aureus *in Western Australia. J Hosp Infect.

[B5] O'Brien FG, Pearman JW, Gracey M, Riley TV, Grubb WB (1999). Community strains of methicillin-resistant *Staphylococcus aureus *involved in a hospital outbreak. J Clin Microbiol.

[B6] Maltezou HC, Giamarellou H (2006). Community-acquired methicillin-resistant *Staphylococcus aureus *infections. Int J Antimicrob Agents.

[B7] Orsi G, Stefano L, Noah N (2002). Hospital-acquired, laboratory-confirmed bloodstream infections : increased hospital stay and direct costs. Infect Control Hosp Epidemiol.

[B8] Cosgrove SE, Qi Y, Kaye KS, Harbarth S, Karchmer AW, Carmeli Y (2005). The impact of methicillin resistance in *Staphylococcus aureus *bacteremia on patient outcomes: mortality, length of stay, and hospital charges. Infect Control Hosp Epidemiol.

[B9] Sanches IS, Saravaiva ZC, Tendeiro TC, Serra JM, Dias DC, de Lencastre H (1998). Extensive intra-hospital spread of a methicillin-resistant *Staphylococcus aureus *clone. Int J Infect Dis.

[B10] Mato R, Santos Sanches I, Venditti M, Platt DJ, Brown A, Chung M, de Lencastre H (1998). Spread of the multiresistant Iberian clone of methicillin-resistant *Staphylococcus aureus *(MRSA) to Italy and Scotland. Microb Drug Resist.

[B11] Lyon BR, Skurray R (1987). Antimicrobial resistance of *Staphylococcus aureus *: genetic basis. Microbiol Rev.

[B12] Grubb WB (1999). Genetics of MRSA. Rev Med Microbiol.

[B13] Blok HE, Troelstra A, Kamp-Hopmans TE, Gigengack-Baars AC, Vandenbroucke-Grauls CM, Weersink AJ, Verhoef J, Masscini EM (2003). Role of healthcare workers in outbreaks of methicillin-resistant *Staphylococcus aureus *: 10-year evaluation from a Dutch university hospital. Infect Control Hosp Epidemiol.

[B14] (1993). National Committee for Clinical Laboratory Standards. Performance for disk diffusion susceptibility tests. Approved Standards.

[B15] (1991). A guide to sensitivity testing: Report of the working party on Antibiotic sensitivity testing of the British Society for Antimicrobial Chemotherapy. Journal Antimicrob Chemother.

[B16] Emslie KR, Townsend DE, Bolton S, Grubb WB (1985). Two distinct resistance determinants to nucleic acid binding compounds in *Staphylococcus aureus*. FEMS Microbiol Lett.

[B17] Al-Haddad AM, Udo EE, Mokadas EM, Sanyal SC, Grubb WB (2001). Persistence of a clone of methicillin-resistant *Staphylococcus aureus *in a burns unit. J Med Microbiol.

[B18] Udo EE, Jacob LE (1998). Conjugative transfer of high-level mupirocin resistance and the mobilization of non-conjugative plasmids in *Staphylococcus aureus*. Microb Drug Resist.

[B19] Hiramatsu K, Hanaki H, Ino T, Yabuta K, Oguri T, Tenover FC (1997). Methicillin-resistant *Staphylococcus aureus *clinical strain with reduced Vancomycin susceptibility. J Antimicrob Chemother.

[B20] Li F, Park SY, Ayers TL, Miller FD, MacFadden R, Nakata M, Lee MC, Effler PV (2005). Methicillin-resistant *Staphylococcus aureus*, Hawaii, 2000–2002. Emerg Infect Dis.

[B21] Panlilio AL, Culver DH, Gaynes RP, Banerjee S, Henderson TS, Tolson JS, Martone WJ (1992). Methicillin- resistant *Staphylococcus aureus *in US hospitals. Infect Control Hosp Epidemiol.

[B22] Kerr S, Kerr GE, Mackintosh CA, Marples RR (1990). A survey of methicillin-resistant *Staphylococcus aureus *affecting patients in England and Wales. J Hosp Infect.

[B23] Seal JB, Moreira B, Bethel CD, Daum RS (2003). Antimicrobial resistance in *Staphylococcus aureus *at a University of Chicago hospitals. A 15 year longitudinal assessment in a large university based hospital. Infect Control Hosp Epidemiol.

[B24] Lee S-O, Cho YK, Kim SY, Lee ES, Park SY, Seo YH (2004). Comparison of trends of resistance rates over 3 years calculated from results for all isolates and for the first isolate of a given species from a patient. J Clin Microbiol.

[B25] Givney RA, Vickery A, Holiday A, Pegler M, Ben R (1998). Evolution of an endemic methicillin-resistant *Staphylococccus aureus *populations in an Australian hospital from 1967–1996. J Clin Microbiol.

[B26] Hsueh PR, Chen WH, Teng LJ, Luh KT (2005). Nosocomial infections due to methicillin-resistant *Staphylococcus aureus *and vancomycin-resistant enterococci at a university hospital from 1991 to 2003: resistance trends, antibiotic usage and *invitro *activities of newer antimicrobial agents. Int J Antimicrob Agents.

[B27] Nimmo GR, Bell JM, Mitchell D, Gosbel IB, Pearman JW, Turnidge J (2003). Antimicrobial resistance in *Staphylococcus aureus *in Australian teaching hospitals, 1989–1999. Microb Drug Resist.

[B28] Grubb WB, Townsend DE, Ashdown N, Tjia T, McGlashan C, Leng T (1986). Genetic analysis of methicillin-resistant *Staphylococcus aureus *from Singapore hospitals. Eur J Clin Microbiol Infect Dis.

[B29] Gillespie MT, Lyon BR, Skurray RA (1990). Typing of methicillin-resistant *Staphylococcus aureus *by antibiotic resistance phenotypes. J Med Microbiol.

[B30] Andrew J, Ashby J, Jevons G, Marshall T, Lines N, Wise W (2000). A comparison of antimicrobial resistance rates in Gram-positive pathogens isolated in the UK from October 1996 to January 1997 and October 1997 to January 1998. J Antimicrob Chemother.

[B31] Zinn CS, Westh H, Rosdahl VT, and the SARISA Study group (2004). An international multicenter study of antimicrobial resistance and typing of hospital *Staphylococcus aureus *isolates from 21 laboratories in 19 countries. Microb Drug Resist.

[B32] Turnidge J, Collignon P (1999). Resistance to fusidic acid. J Antimicrob Agents.

[B33] Mulvey MR, MacDougal L, Cholin B, Horsman G, Fidyk M, Woods S, and the Saskatchewan CA-MRSA Study Group (2005). Community-associated methicillin-resistant *Staphylococcus aureus*, Canada. Emeg Infect Dis.

[B34] Udo EE, Jacob LE (2000). Characterisation of methicillin-resistant *Staphylococcus aureus *from Kuwait hospitals with high-level fusidic acid resistance. J Med Microbiol.

[B35] Udo EE, Al-Sweih N, Noronha B (2006). Characterization of non-multiresistant methicillin-resistant *Staphylococcus aureus *(including EMRSA-15) in Kuwait's Hospitals. Clin Microbiol Infect.

[B36] Udo EE, Farook VS, Mokadas EM, Jacob LE, Sanyal SC (1999). Molecular fingerprinting of mupirocin-resistant methicillin-resistant *Staphylococcus aureus *from a Burns unit. Int J Infect Dis.

[B37] Udo EE, Jacob LE, Mathew B (2001). Genetic analysis of methicillin-resistant *Staphylococcus aureus *expressing high- and low- level mupirocin resistance. J Med Microbiol.

[B38] Udo EE, A-Sweih N, Mohanakrihnan S, West PWJ (2006). Antibacterial resistance and molecular typing of methicillin-resistant *Staphylococcus aureus *in a Kuwaiti general hospital. Medical Priciples and Practice.

[B39] Walker ES, Levy F, Shorman M, David G, Abdalla J, Sarubbi FA (2004). A decline in mupirocin resistance in methicillin-resistant *Staphylococcus aureus *accompanied administrative control of prescriptions. J Clin Microbiol.

